# Signaling of Macrophage Inflammatory Protein (MIP)-3β Facilitates Dengue Virus-Induced Microglial Cell Migration

**DOI:** 10.3390/v10120690

**Published:** 2018-12-05

**Authors:** Ming-Kai Jhan, Ting-Jing Shen, Po-Chun Tseng, Yung-Ting Wang, Chiou-Feng Lin

**Affiliations:** 1 Graduate Institute of Medical Sciences, College of Medicine, Taipei Medical University, Taipei 110, Taiwan; williamjhan2730@gmail.com (M.-K.J.); bibobibo410@hotmail.com (T.-J.S.); 2 Department of Microbiology and Immunology, School of Medicine, College of Medicine, Taipei Medical University, Taipei 110, Taiwan; iluc0720@hotmail.com (P.-C.T.); olivia760717@gmail.com (Y.-T.W.); 3 Center of Infectious Disease and Signaling Research, National Cheng Kung University, Tainan 701, Taiwan

**Keywords:** dengue virus, microglia, migration, caveolae, MIP-3β

## Abstract

The infection by dengue virus (DENV) of microglia causes cell activation and migration via a mechanism involving viral entry, RNA release, and Toll-like receptor 3 signaling. In this study, we demonstrated that secreted chemotactic factors present in microglial conditioned medium (MCM) facilitated cell motility in the murine BV2 microglial cells. The pharmacological disruption of lipid rafts/caveolae reduced DENV- and ultraviolet (UV)-inactivated MCM-induced microglial cell migration. An antibody-based cytokine/chemokine array showed an increase in macrophage inflammatory protein (MIP)-3β in MCM produced using DENV-infected cells. The pharmacological inhibition of c-Jun N-terminal kinase (JNK) retarded UV-MCM-induced microglial cell migration. These results demonstrate that secreted MIP-3β and its effect on the JNK signaling pathways mediates DENV-induced BV2 microglial cell migration.

## 1. Introduction

Infections caused by all four dengue virus (DENV) serotypes cause dengue fever and severe dengue, arthropod-borne viral diseases that are endemically reported in the Eastern Mediterranean, American, South-East Asian, Western Pacific and African regions [1]. Dengue fever is predominantly characterized in dengue patients by clinical signs that include the presence of a fever, rash, and headache. In some cases, DENV infection may induce severe dengue in patients, as revealed by the presence of many indicators, including plasma leakage, bleeding, loss of consciousness, severe gastrointestinal and organ impairment, and other unusual manifestations [1,2]. In these patients with severe dengue, the prospective lethality should be determined. However, this issue remains a struggle for physicians and scientists regarding the complicated pathogenesis in DENV infection and of dengue disease progression [3,4]. Antiviral drugs and preventive vaccines are currently being tested in several clinical trials and experimental animal models [5,6].

Although rare, severe dengue patients may also exhibit neurological complications, including dengue encephalopathy, encephalitis, neuromuscular complications, and neuro-ophthalmic involvement [7]. With an undefined pathogenesis of CNS infections, it is hypothesized that DENV infection of the brain may cause direct and indirect effects on neurotoxicity and brain dysfunction, as the viral genome, proteins, and particles can be detected in the brains of fatal dengue patients [8,9]. In experimentally infected mice, DENV was reported to infect neuronal cells and microglia in the infected brains following blood–brain barrier (BBB) destruction and neurotoxicity, similar to viral encephalitis-like symptoms [10,11,12,13]. In vitro and in vivo studies have demonstrated the infectivity of DENV in neuronal cells, which was directly associated with neurotoxicity [11,12,13,14,15]. Based on these findings, it has been postulated that neurotrophic DENV causes CNS infections [11,12,13].

The DENV-mediated activation of microglia, the resident macrophage-like immune cells in the brain, is speculated to amplify neuroinflammation and antiviral immunity [12,16]. We previously demonstrated the infectivity of DENV in microglial cells in vitro and in vivo [12,17]. In microglia, DENV was internalized in endosomes via clathrin-mediated endocytosis to release viral RNA for replication following endosomal acidification [17]. In DENV-infected microglial BV2 cells, we further observed that clathrin-mediated endocytosis followed by Toll-like receptor (TLR) 3 activation induces an increase in microglial cell migration. In this study, we investigated the molecular mechanisms involved in these processes and further demonstrated the involvement of the secreted chemotactic factors in indirectly facilitating DENV-induced BV2 microglial cell motility.

## 2. Materials and Methods

### 2.1. Reagents

The reagents and antibodies (Abs) used in this study included the following: the lipid rafts/caveolae inhibitor nystatin (Sigma-Aldrich Co., St. Louis, MO, USA); the c-Jun N-terminal kinase (JNK) inhibitor SP600125 (Calbiochem, San Diego, CA, USA); and the neutralizing anti-MIP-3β (C-C motif chemokine ligand 19, CCL19) (R&D systems, Minneapolis, MN, USA). All drug treatments were assessed for their ability to cause cytotoxic effects using cytotoxicity assays prior to the experiments. Noncytotoxic dosages were used in this study.

### 2.2. Cell Culture

BV2 immortalized murine microglial cells, obtained from Dr. C. C. Huang (Department of Pediatrics, National Cheng Kung University, Tainan, Taiwan), were grown in Dulbecco’s modified Eagle’s medium (DMEM) supplemented with 10% heat-inactivated fetal bovine serum (FBS) (Sigma-Aldrich), 50 U/mL of penicillin and 50 μg/mL of streptomycin and were incubated under a humidified atmosphere with 5% CO_2_ and 95% air. Baby hamster kidney (BHK)-21 cells (ATCC^®^ CCL-10™) and *Aedes albopictus* clone C6/36 cells (ATCC^®^ CRL-1660™) were cultured in DMEM (Invitrogen Life Technologies, Carlsbad, CA, USA) containing FBS.

### 2.3. Virus Culture

DENV serotype 2 (DENV2 PL046) was maintained in C6/36 cells, with the virus-containing supernatant concentrated according to previously described procedures [12] and stored at −80 °C prior to use. The virus titer was determined via a previously described plaque assay [12] using the BHK-21 cell line, which allowed for a concentration of 10^7^ pfu/mL to be achieved from the preparation.

### 2.4. DENV Infection

BV2 cells (10^5^ cells per well) were seeded in 6-well plates and incubated overnight, followed by the addition of DMEM containing DENV at an multiplicity of infection (MOI) of 50. The cells were incubated for 90 min at 37 °C, after which they were washed once with medium and then incubated at 37 °C under an atmosphere containing 5% CO_2_. Following the infection, the viral supernatants in microglial conditioned medium (MCM) were collected at different time points and checked using plaque assays. For UV irradiation-mediated DENV inactivation, MCM was exposed to a 15 W UV lamp at a distance of 10 cm for 1.5 h.

### 2.5. Cell Viability and Cytotoxicity

Assays were performed using a Cell Proliferation kit I (MTT) (Sigma-Aldrich) and a Cytotoxicity Detection kit (LDH) (Roche Diagnostics, Lewes, UK) to detect cell viability and cytotoxicity, respectively, according to the manufacturer’s instructions.

### 2.6. Wound-Healing Assay

A scratch wound was created using a Culture-Insert (BD Labware Europe, Le Pont De Claix, France) following the procedures described in our previous study [17]. The wound was photographed at 0 and 12 h post-infection, and the cells that migrated into the cleared wound area were counted using ImageJ (http://imagej.nih.gov/ij/download.html) and the reduction of the wound area was measured.

### 2.7. Cytokine Antibody Array

MCM was stored at −80 °C. A cytokine antibody array (Mouse Cytokine Antibody Array III M0308003; RayBiotech, Inc., Norcross, GA, USA) containing 62 different cytokine antibodies was used to measure the profile of cytokine production according to the manufacturer’s instructions. Following detection, 45/62 cytokines/chemokines displaying signals were calculated and shown by mean pixel density.

### 2.8. Statistical Analysis

Data obtained from three independent experiments were presented as the means ± standard deviation (SD) followed by statistical analysis by using Prism version 5 (GraphPad Software, San Diego, CA, USA). An unpaired Student’s *t*-test and one-way ANOVA with Tukey’s multiple comparison post hoc tests were performed, and significant differences were observed at *p* < 0.05.

## 3. Results

### 3.1. Conditioned Medium from DENV-Infected BV2 Microglia Cultures Stimulates Cell Migration

**Figure 1:**
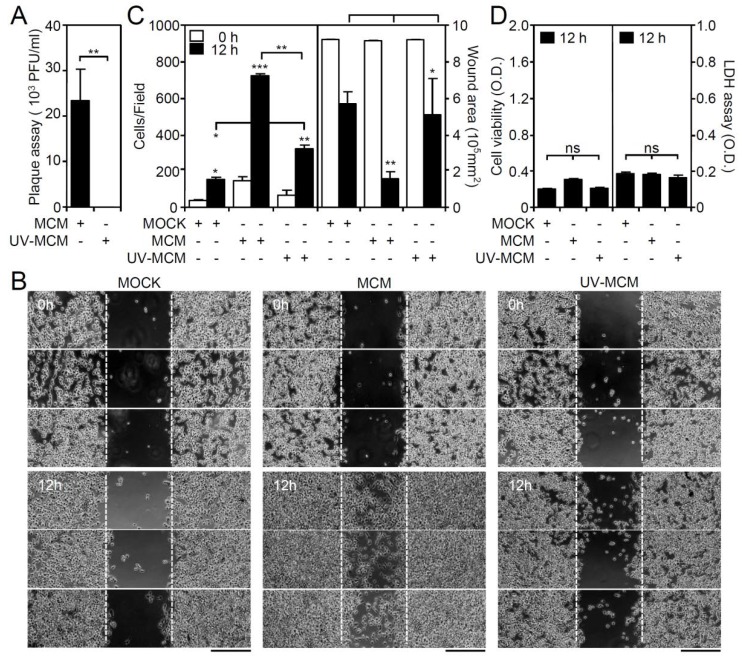
Microglial conditioned medium (MCM) collected from dengue virus (DENV)-infected BV2 microglia stimulates cell migration. BV2 cells were inoculated with DENV 2 (MOI = 50) for 12 h, and the MCM was collected in the absence or presence of UV irradiation. (**A**) Plaque assay confirmation of viral release. (**B**) A wound-healing assay performed in triplicate showed cell migration. Scale bar, 200 µm. (**C**) Image analysis was performed using ImageJ based on the number of migrating cells and the changes in the wound area. The quantitative data are depicted as the means ± SD. (**D**) MTT and LDH assays showed the cell viability and cytotoxicity, respectively, of BV2 cells inoculated with MCM and UV-inactivated MCM. The quantitative data (optical density, O.D.) are shown as the means ± SD from three independent experiments. * *p* < 0.05, ** *p* < 0.01, and *** *p* < 0.001. ns, not significant.

Our previous studies showed that DENV infection increases microglial cell migration through the TLR3-regulated pathway [17]. To investigate the roles of common chemotactic factors generally involved in cell migration, microglial conditioned medium (MCM) was collected at 12 h post-infection and tested for its effect on cell motility. To eliminate free DENV from the MCM, ultraviolet (UV)-mediated DENV inactivation was performed and confirmed by a plaque assay (Figure 1A). In a wound-healing assay (Figure 1B), the treatment of wounds with MCM significantly (*p* < 0.01) induced microglial cell migration, as assessed by measuring the increase in the number of migrated cells and the decrease in the healing wound area (Figure 1C). In contrast, as compared with the MCM-treated group, wounds treated with UV-inactivated MCM (UV-MCM) showed a decrease in microglial cell motility. However, wounds treated with UV-MCM still significantly (*p* < 0.01) enhanced motility in microglial cells as compared with the mock control (MOCK). Without effects on cell viability and cytotoxicity (Figure 1D), these results indicate that either DENV or soluble factors secreted from DENV-infected microglia synergistically promote microglial cell migration.

### 3.2. Blocking Lipid Rafts/Caveolae Retards DENV-Induced BV2 Microglial Cell Migration

**Figure 2:**
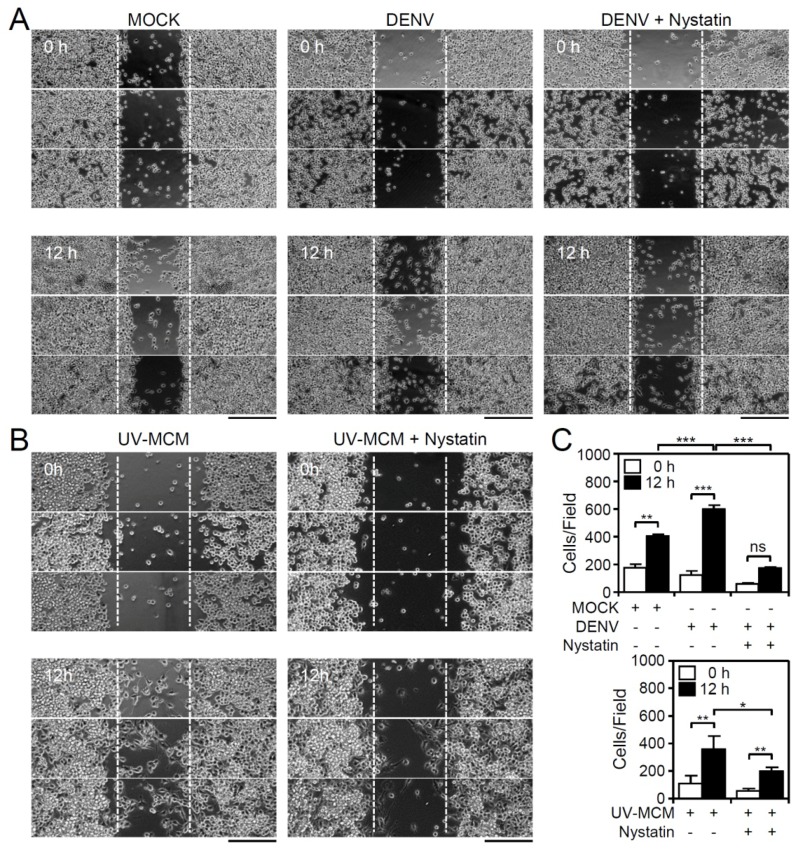
Inhibition of lipid rafts/caveolae retards DENV-induced BV2 microglial cell migration. Migration in DENV- and UV-MCM-stimulated BV2 cells incubated with or without lipid rafts/caveolae inhibitor (Nystatin, 5 µM) treatment was measured by the wound-healing assay 12 h post-infection. (**A**,**B**) The wound-healing assay and (**C**) the measurement of the migrating cell number showed cell migration. Scale bar, 200 µm. All quantitative data are shown as the means ± SD from three independent experiments. * *p* < 0.05, ** *p* < 0.01, and *** *p* < 0.001. ns, not significant.

In general, autocrine and/or paracrine-secreted chemotactic factors are essential for triggering cell migration following their internalization with chemokine receptors, which probably occurs via the clathrin-mediated pathway and by lipid rafts/caveolae-dependent routes [18]. Clathrin-mediated endocytic trafficking is required for DENV internalization in BV2 microglia, and the pharmacological inhibition of clathrin-mediated endocytosis reduces the DENV-induced increase in microglial migration [17]. Additionally, treatment of cells with nystatin, an inhibitor of lipid rafts/caveolae, also significantly (*p* < 0.01) reduced both the DENV- (Figure 2A,C) and UV-MCM-induced increase (Figure 2B,C) in microglial cell migration. However, no further effects were caused by nystatin on the basal level of cell migration in mock-treated cells (Supplementary File 1). These results indicate the involvement of lipid rafts/caveolae-mediated signaling for promoting microglial cell migration.

### 3.3. Screening Cytokines/Chemokines in DENV-Infected BV2 Microglial Cells

**Figure 3:**
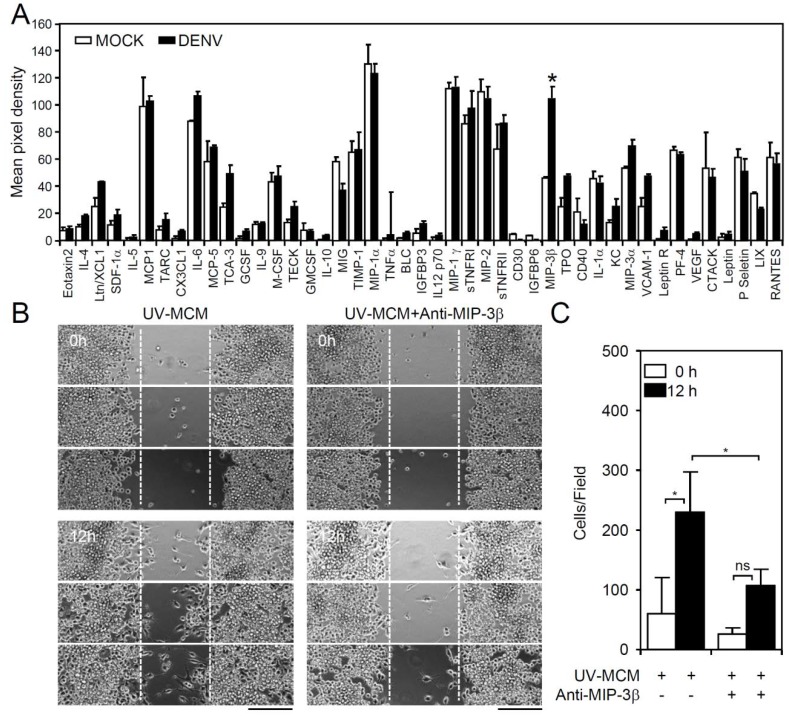
DENV infection induces expression of cytokines/chemokines in BV2 microglial cells. MCM of BV2 cells infected with DENV were collected. (**A**) A membrane antibody array containing 62 different cytokine antibodies was utilized to detect the secretion of soluble factors. The values are shown as the mean pixel density. (**B**,**C**) The wound-healing assay and the measurement of the migrating cell number showed cell migration in UV-MCM-stimulated BV2 cells incubated with or without neutralizing anti-macrophage inflammatory protein (MIP)-3β (CCL19) (4 µg/mL) treatment 12 h post-stimulation. Scale bar, 200 µm. All quantitative data are shown as the means ± SD from three independent experiments. * *p* < 0.05. ns, not significant.

Under TLR3 signaling, the phosphoinositide 3-kinase (PI3K), extracellular signal-regulated kinase (ERK), and nuclear factor kappa-light-chain-enhancer of activated B cells (NF-κB)-regulated signaling pathways are required for mediating microglial cell migration [17]. Both direct (infection-dependent) and indirect (post-infection-regulated) signaling are hypothesized to be involved in this process. To evaluate the possible chemotactic factors involved in DENV-induced microglial cell migration, a membrane-based antibody array was performed to screen the expression levels of 62 cytokines/chemokines in DENV-infected cells [12]. Following DENV infection of microglial cells for 12 h, MCM was collected, and the resulting antibody array data only showed a significant increase (*p* < 0.05) in the levels of the chemokine macrophage inflammatory protein (MIP)-3β (chemokine (C-C motif) ligand CCL19) (Figure 3A). To validate the importance of MIP-3β (CCL19), the blocking approach was performed by using a neutralizing antibody. Wound healing assay showed that the administration of anti-MIP-3β (CCL19) significantly (*p* < 0.05) decreased MCM-induced cell migration (Figure 3B,C). These results demonstrated the induction of chemokine MIP-3β (CCL19) expression in DENV-infected microglial cells.

### 3.4. A MIP-3β/c-Jun N-Terminal Kinase (JNK) Signaling Pathway Mediates DENV-Induced BV2 Microglial Cell Migration

**Figure 4:**
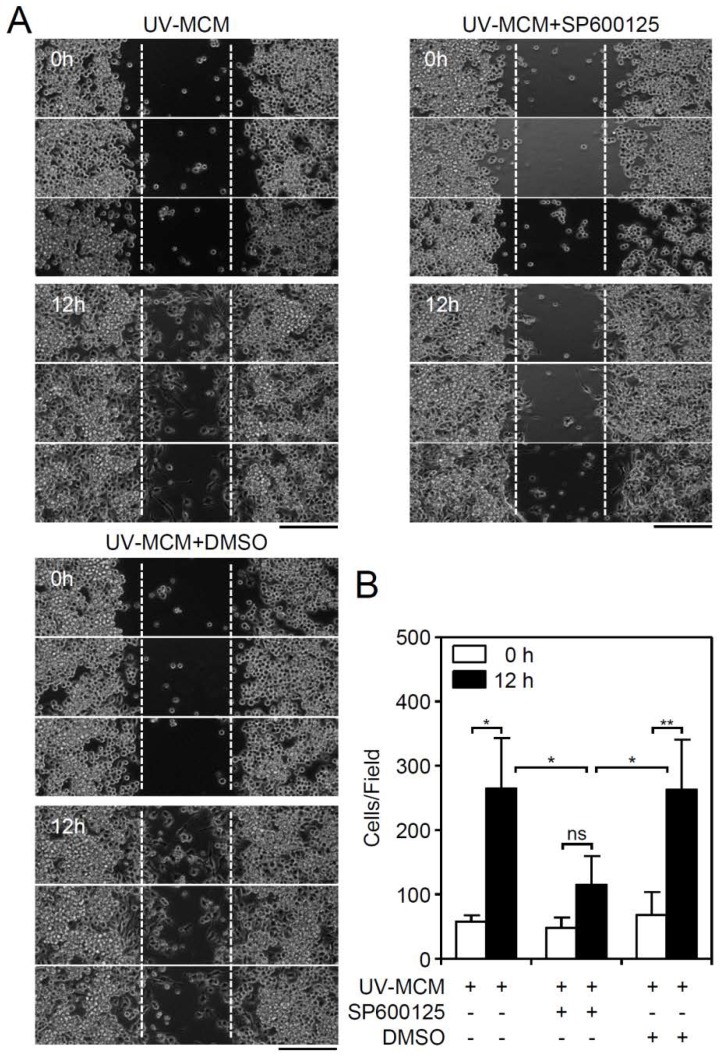
Pharmacological inhibition of JNK reduces DENV-induced BV2 microglial cell migration. Migration in UV-MCM-stimulated BV2 cells incubated with or without a JNK inhibitor (SP600125, 20 µM) treatment was measured by the wound-healing assay 12 h post-infection. (**A**) The wound-healing assay and (**B**) the measurement of the migrating cell number showed cell migration. Dimethyl sulfoxide (DMSO) as a solvent control. Scale bar, 200 µm. All quantitative data are shown as the means ± SD from three independent experiments. * *p* < 0.05 and ** *p* < 0.01. ns, not significant.

MIP-3β (CCL19)-mediated cell migration involves its activation of JNK, causing MIP-3β (CCL19)-induced endocytosis [19], which regulates cellular chemotaxis and migration. To confirm the possible involvement of these molecular mechanisms, pharmacological inhibition of JNK by SP600125 was performed, which significantly (*p* < 0.01) reduced the UV-MCM-induced increase in microglial cell migration (Figure 4A,B). These results show that JNK signaling is required for cell migration in DENV-infected microglial cells.

## 4. Discussion

DENV infection may cause neurological complications in severe dengue patients. Although it is a rare situation, the underlying mechanism of this condition remains undefined. Because of the neurotoxic effects resulting from neuronal cell injury, CNS inflammation following DENV infection that causes viral encephalitis-like symptoms may also be involved. In general, active microglia, the resident macrophages in the brains, are the major immune cells in the brain responsible for immunosurveillance and for responding to virus-induced neuropathology [20,21]. We previously observed that DENV not only infects microglial cells in vitro and in vivo but also induces cell morphological changes toward a migratory state [17]. Image analysis of a wound healing assay further confirmed the increased motility of DENV-infected microglial cells. The induction of microglial cell motility by DENV infection, at least in part, is regulated by clathrin-mediated endocytosis, dsRNA signaling, and TLR3 activation. Interestingly, previous reports demonstrated a pro-inflammatory role [16] and antiviral response [12] elicited by DENV-infected microglia. An increase in microglial cell motility is speculated to contribute to CNS inflammation and an increased immune response.

To investigate the possible mechanisms for DENV-induced microglial cell migration, we previously showed that UV-inactivated DENV completely lost its infectivity and the ability to promote cell motility, indicating that DENV infectivity causes these effects [17]. In this study, we collected MCM from DENV-infected microglial cells and used UV irradiation to inactivate the infectivity of free DENV in MCM. Our data further demonstrate a possible synergistic effect of soluble factors secreted from DENV-infected microglia in facilitating microglial cell motility. In DENV-infected dendritic cells, viral infection also causes migratory effects on dendritic cells in a galectin-9- and interferon-lambda1-regulated manner [22,23]. Infected cells migrate toward the chemoattractants MIP-3β (CCL19) and CCL21. By using a cytokine/chemokine screening approach, an increase in MIP-3β (CCL19) production can be observed in DENV-infected microglial cells. Other undetected factors related to cell migration, which could be synergistically co-operated with MIP-3β (CCL19), need further validation. The results indicate that both DENV and the possible chemokine MIP-3β (CCL19) released from infected cells may synergistically promote microglial cell migration. C-C chemokine receptor (CCR) 7, a receptor for MIP-3β (CCL19) binding, is also expressed in microglial cells [24] and is induced in mature dendritic cells [25]. The results of this study and our previous works [17] suggest that DENV infection in microglial cells increases cell motility.

**Figure 5:**
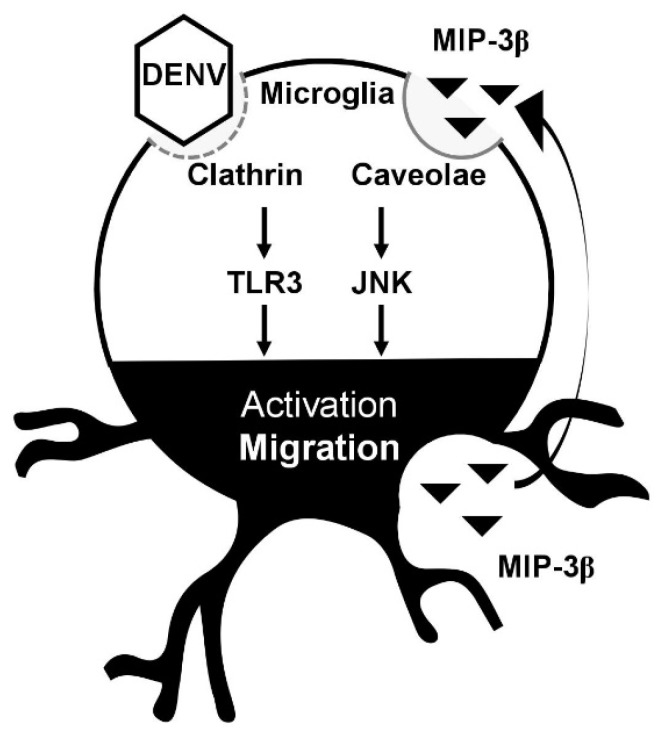
A hypothetical model of DENV-induced microglial cell migration. Secreted chemotactic factors in DENV-infected microglia facilitate cell motility. Pharmacologically disrupting lipid rafts/caveolae reduces microglial cell migration. MIP-3β is secreted from DENV-infected microglia. Targeting JNK retards microglial cell migration.

Our results also demonstrate that the inhibition of lipid rafts/caveolae reduces DENV- and MCM-induced cell migration. Because we previously showed that the endocytosis of DENV via a clathrin-mediated pathway is required for DENV-induced cell migration [17], the signaling of lipid rafts/caveolae for microglial cell migration is therefore speculated to be important for secreted MIP-3β (CCL19). Importantly, the migratory effect of MIP-3β (CCL19) and its receptor CCR7 is activated in a lipid raft/caveolae-dependent manner [26]. Thus, our data support the MIP-3β (CCL19)/CCR7-mediated signaling of lipid rafts/caveolae for regulating microglial cell migration. Consistent with previous observations that MIP-3β (CCL19) causes cell migration by activating JNK [19], our data confirmed the requirement of such signaling pathways in facilitating DENV-induced BV2 microglial cell migration. Furthermore, other signaling molecules required for mediating either MIP-3β (CCL19)/CCR7- or TLR3-induced cell migration need further investigation. In conclusion, as summarized in Figure 5, our current findings not only demonstrate a DENV-induced increase in BV2 microglial cell migration but also identify the possible processes for mediating such effects via clathrin-mediated endocytosis, dsRNA replication, and TLR3 signaling [17]. This study further demonstrates the underlying mechanism for activating BV2 microglial cell migration involving the secretion of MIP-3β (CCL19) and its signaling pathway. The findings of this study need to be validated either in vitro by using primary microglia or in vivo by using an animal model.

## Supplementary Materials

The following are available online at http://www.mdpi.com/1999-4915/10/12/690/s1.
