# Supplementary figures and images for: Signaling of Macrophage Inflammatory Protein (MIP)-3β Facilitates Dengue Virus-Induced Microglial Cell Migration
Source: Viruses. 2018 Dec 5;10(12):690. doi: 10.3390/v10120690 (PMC6316022; doi:10.3390/v10120690)

Supplementary File 1

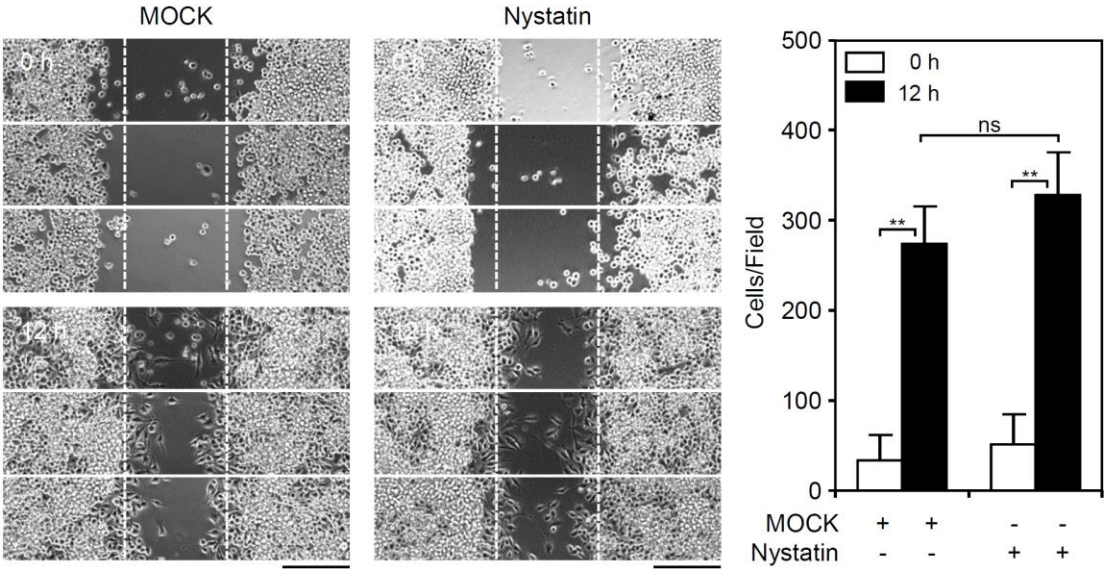

Supplement: Supplementary file 1 [file viruses-10-00690-s001.pdf]
